# Diversity of Cultivable Midgut Microbiota at Different Stages of the Asian Tiger Mosquito, *Aedes albopictus* from Tezpur, India

**DOI:** 10.1371/journal.pone.0167409

**Published:** 2016-12-12

**Authors:** Kamlesh K. Yadav, Sibnarayan Datta, Ashok Naglot, Ajitabh Bora, Vanlal Hmuaka, Sameer Bhagyawant, Hemanta K. Gogoi, Vijay Veer, P. Srinivas Raju

**Affiliations:** 1 Biotechnology Division, Defence Research Laboratory, DRDO, Tezpur, India; 2 School of Studies in Biotechnology, Jiwaji University, Gwalior, India; Universidade Federal do Rio de Janeiro, BRAZIL

## Abstract

*Aedes aegypti* and *Ae*. *albopictus* are among the most important vectors of arboviral diseases, worldwide. Recent studies indicate that diverse midgut microbiota of mosquitoes significantly affect development, digestion, metabolism, and immunity of their hosts. Midgut microbiota has also been suggested to modulate the competency of mosquitoes to transmit arboviruses, malaria parasites etc. Interestingly, the midgut microbial flora is dynamic and the diversity changes with the development of vectors, in addition to other factors such as species, sex, life-stage, feeding behavior and geographical origin. The aim of the present study was to investigate the midgut bacterial diversity among larva, adult male, sugar fed female and blood fed female *Ae*. *albopictus* collected from Tezpur, Northeastern India. Based on colony morphological characteristics, we selected 113 cultivable bacterial isolates for 16S rRNA gene sequence based molecular identification. Of the 113 isolates, we could identify 35 bacterial species belonging to 18 distinct genera under four major phyla, namely Proteobacteria, Firmicutes, Actinobacteria and Bacteroidetes. Phyla Proteobacteria and Firmicutes accounted for majority (80%) of the species, while phylum Actinobacteria constituted 17% of the species. Bacteroidetes was the least represented phylum, characterized by a single species- *Chryseobacterium rhizoplanae*, isolated from blood fed individuals. Dissection of midgut microbiota diversity at different developmental stages of *Ae*. *albopictus* will be helpful in better understanding mosquito-borne diseases, and for designing effective strategies to manage mosquito-borne diseases.

## Introduction

Among the insects, mosquitoes play a significant role in transmission of various diseases like Dengue, Zika fever, Chikungunya, Yellow fever, Malaria, Japanese encephalitis, lymphatic filariasis etc. Of the medically important mosquito species, *Aedes aegypti* and *Ae*. *albopictus* are the most important vectors of arboviruses, including Zika virus (ZIKV), Dengue virus (DENV), Yellow fever virus (YFV), and Chikungunya virus (CHIKV) [1–6]. In the recent decades, the burden of Dengue has dramatically increased and approximately 40% of the population in 100 countries is estimated to be affected. The incidences are very high, especially in the developing nations of South-East Asia, Western Pacific, and Americas [7]. The recent outbreaks of Zika fever and associated comorbidities have attested to the increasing risk of vector-borne emerging and re-emerging diseases, worldwide [8–9].

Recent metagenomic studies on mosquito midgut have revealed the presence of a diverse microbiota, which can significantly affect the development, digestion, metabolism, immunity and other physiological functions of their hosts. This midgut microbiota have also been suggested to alter the competency of mosquitoes to transmit pathogens like arboviruses, malaria parasite etc [10–12]. A number of bacterial species isolated from the mosquito midguts have also been utilized for manipulating their midgut microbiota (paratransgenesis), to modulate the vector competency of the mosquitoes, as a potent strategy for vector management [13–15]. Interestingly, the midgut microbial flora is dynamic and the diversity changes with the development of the vectors, in addition to other factors such as species, sex, life-stage, feeding behavior and geographical origin [16–22]. As a conventional assumption, midgut bacterial population is thought to be acquired from the environment, in which the said species develops. The first acquisition of bacteria occurs during the larval stages and the subsequent acquisition occurs during the adult stages [11]. Apart from the environment, the feeding habit also significantly influences the microbial diversity of the vectors. Adult mosquitoes prefer nectar as their first meal, resulting in increase of carbohydrate in the midgut, while the level of protein increases significantly after the blood meal in female mosquitoes. This change from high carbohydrate levels to protein dramatically modulates the midgut environment, inducing a shift in the midgut microbiome [23–28]. Very interestingly, microbial diversity reduces post-blood meal, signified by increase in enteric bacterial population, to counter the increased oxidative and nitrosative stresses associated with the metabolism of blood [28]. Although, midgut microbiota have been studied in a number of different vector mosquito species, but the literature on microbial diversity in different developmental stages of *Aedes* is particularly scanty. In this study, we attempted to explore the midgut microbial diversity of larvae, sugar fed female, blood fed female and male of *Ae*. *albopictus*, collected from Tezpur, a town situated in northeastern India, considered as an important biodiversity hot-spot.

## Materials and Methods

### Sample collection

We collected fourth instar larvae and pupae of *Ae albopictus* mosquitoes from waste tyres, plastic & metal pots from Solmara, Tezpur [26.63°N 92.8°E], India, and transported to the laboratory in pre-sterilized plastic bottles. Collection was done during post monsoon season when the mosquito breeding was at its peak. In the laboratory, field collected samples were sorted and pupae were transferred to pre-sterilized net cages for emergence of adult. Larvae and pupae were incubated in water, collected from the breeding sites.

The samples used in this study were collected from public land, which do not come under the categories of national parks/ protected areas. Furthermore, there was no involvement of collection or disturbance to endangered or protected species, during collection of samples. Therefore, no specific permission was required for samples collection.

### Dissection and isolation of midgut

In the present study, we selected the larvae and adults (males, sugar fed females and blood fed females) for dissection and isolation of midgut bacteria. Initially, we separated 30 fourth instar larvae from the pool for dissection and isolation of bacteria. Subsequently, after emergence, all the adults were provided with 10% sucrose solution [Sigma Aldrich, St. Louis, USA] as a food source. After 24 hrs, we segregated 30 male and female mosquitoes for dissection and isolation of midgut bacteria. To the remaining mosquitoes, we fed blood through biting of rabbit and after 24 hrs, we took out 30 blood fed mosquitoes for dissection. We identified the mosquitoes based on their macroscopic morphological characteristics prior to dissection [29].

All the dissections were performed under *Leica* stereomicroscope (Model: *Leica* EZ4 HD) using sterilized apparatus, according to a protocol described elsewhere [30]. Prior to dissection, all the adult and larvae samples were surface sterilized with 75% ethanol for 5 min, followed by washing with phosphate buffered saline (PBS) twice. All the dissections were carried out in sterile conditions and midgut sections were separately homogenized in 100 μl of PBS [30–31].

### Isolation and purification of midgut bacteria

Gut homogenates were serially diluted (10 folds) with PBS and 100 μl of each dilution was pour-plated on nutrient agar media (Himedia, India) and incubated at 37°C for 24–48 hrs. The last wash (PBS) of larvae and adults was taken as a control, pour plated and incubated as the homogenate of dissected midgut. All the microbiological procedures were carried out in a sterile environment, strictly following aseptic laboratory practices and negative controls (sterile PBS) were included throughout the experiment. We selected morphologically distinct bacterial colonies for subculture on nutrient agar plates and for isolation of pure colonies.

### Genomic DNA isolation and PCR amplification of 16S rRNA gene

For amplification of 16S rRNA gene, genomic DNA was isolated, as mentioned previously [30,32]. In brief, genomic DNA was isolated from freshly cultured bacterial cells, and re-suspended in Tris-EDTA buffer (pH-8). For efficient lysis of bacterial cells, a freezing-thawing step (freezing at -80°C and thawing at 75°C, for 3 cycles) was incorporated, followed by lysozyme and proteinase-K treatment. Genomic DNA was precipitated in isopropanol, DNA pellets were air dried and re-suspended in TE buffer. An amplicon of approximately 1.5 kb was amplified from the small subunit of 16S rRNA gene using primer set 16S1 (5’-GAGTTTGATCCTGGCTCA-3’) and 16S2 (5’-CGGCTACCTTGTTACGACTT-3’) and an automated thermal cycler (BioRad, USA) [33].

### Sequencing and phylogenetic analysis

PCR products were purified (Chromous Biotech, India) and both the strands were directly sequenced on an ABI 3500*xl* Genetic Analyzer (Applied Biosystems Inc. Foster City, CA). Sequences were manually checked, edited, analyzed, and aligned using BioEdit software (*ver*. 7.2) and Chromas Lite (*ver*. 2.1) and were submitted to the GenBank under the accession numbers (KU550135 to KU550185). The sequences obtained in our study were compared with GenBank database using the BLAST algorithm (http://www.ncbi.nlm.nih.gov/BLAST) and the EzTaxon server (http://www.ezbiocloud.net/eztaxon) to search the homologous sequences [34–35]. The homologous sequences were retrieved from the Genbank, and aligned using ClustalW program. Phylogenetic relatedness among 87 sequences (including 36 reference) was determined by tree reconstructed using Neighbor-Joining method (Kimura-2 parameter for distance calculation), incorporated in MEGA 6.0 package [36]. Robustness of the phylogenetic tree was examined through 1000 bootstrap replicates, and the consensus tree was used for analysis.

### Statistical analysis

A non-parametric, Friedman test was performed to estimate the differences in prevalence of bacterial species between sugar fed, blood fed, male and larvae samples of *Ae*. *albopictus* midguts (p < 0.05, 95% confidence interval). Diversity of isolated bacterial species from the midgut of all samples was analyzed using various indices *i*.*e*. Simpson Index [37], Shannon Index, and Evenness [38]. The biodiversity was studied for the determination of diversity richness, evenness and dominance of obtained bacterial species from each sample categories [31]. Good’s coverage was calculated by using the formula (1-n/N)* 100, where n represents a single bacterial isolate and N denotes total bacterial isolates from one mosquito species [39].

## Results

**Fig 1:**
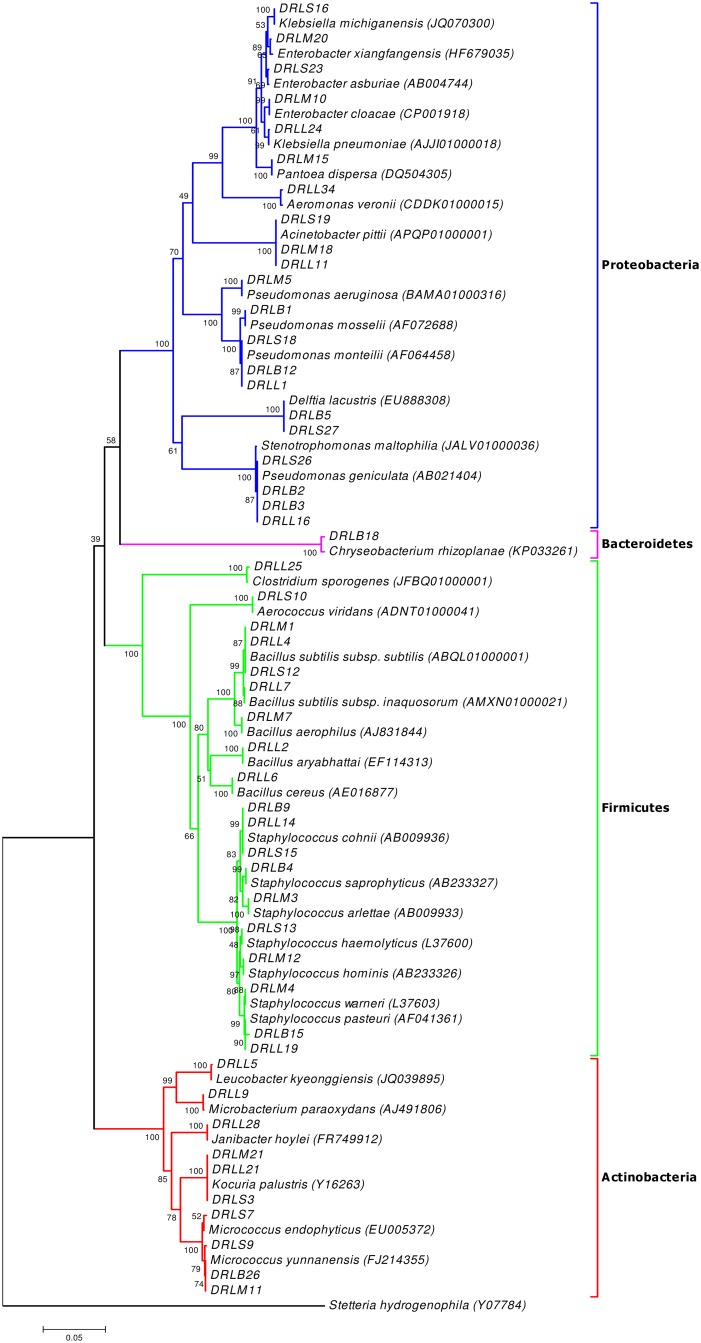
Phylogenetic analysis of bacterial isolates from midgut of *Ae*. *albopictus*. Phylogenetic tree based on partial 16S rRNA gene sequences, reconstructed through neighbor joining algorithm using Kimura 2 distance parameter method. The percentage bootstrap values obtained with 1000 replications are denoted at branch node.

With an aim to examine the diversity of midgut microbiota in various life stages of *Ae*. *albopictus*, we collected and isolated cultivable bacteria from the midgut of larvae, male, sugar fed female and blood fed female mosquitoes, collected from Tezpur, Assam. Based on colony morphological characteristics, we selected 113 bacterial isolates for 16S rRNA gene sequence based identification. From all the categories of individuals, we could identify 35 distinct bacterial species from 18 genera, which belonged to four major phyla namely Proteobacteria (40.0%), Firmicutes (40.0%), Actinobacteria (17.14%), and Bacteroidetes (2.86%) (Fig 1 and S1 Table).

All identified bacterial species from all the categories of individuals were classified in 14 different families in which, Staphylococcaceae (20.00%) was most abundant, followed by Enterobacteriaceae (17.14%), Bacillaceae (14.29%), Pseudomonadaceae (8.57%), Micrococcaceae (8.57%), Microbacteriaceae (5.71%), Xanthomonadaceae (5.71%), Aerococcaceae (2.86%), Aeromonadaceae (2.86%), Clostridiaceae (2.86%), Comamonadaceae (2.86%), Flavobacteriaceae (2.86%), Intrasporangiaceae (2.86%), Moraxellaceae (2.86%) (S1 Table). The bacterial species belonging to families Micrococcaceae, Pseudomonadaceae, Staphylococcaceae were identified from all the categories of the individuals (Larvae, male, sugar fed and blood fed) while, species belonging to family Aerococcaceae could be isolated only from sugar fed individuals, Flavobacteriaceae from blood fed and Microbacteriaceae, Aeromonadaceae, Clostridiaceae, Intrasporangiaceae from larvae.

The most abundant bacterial species in the sugar fed females and larvae individuals was *Acinetobacter pittii* with an abundance of 22.22% and 14.71% respectively, while *Pseudomonas monteilii* (19.35%) was the most abundant species in the blood fed individuals, and *Pantoea dispersa* (19.04%) in the adult males. All these bacterial species belonged to the phylum Proteobacteria. The phylum Bacteroidetes was represented by only a single bacterial species, *Chryseobacterium rhizoplanae*, isolated and identified only from blood fed individuals.

### Distribution of bacterial species in different stages

#### Bacterial isolates from midgut of sugar fed *Ae*. *Albopictus*

**Fig 2:**
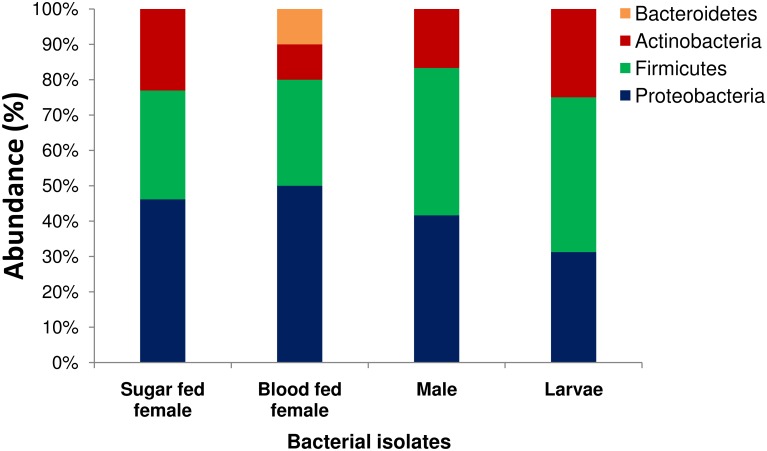
Abundance of identified bacterial species belonging to their respective phylum isolated from the midgut of adult sugar fed female, blood fed female, male, and larvae of *Aedes albopictus*.

**Fig 3:**
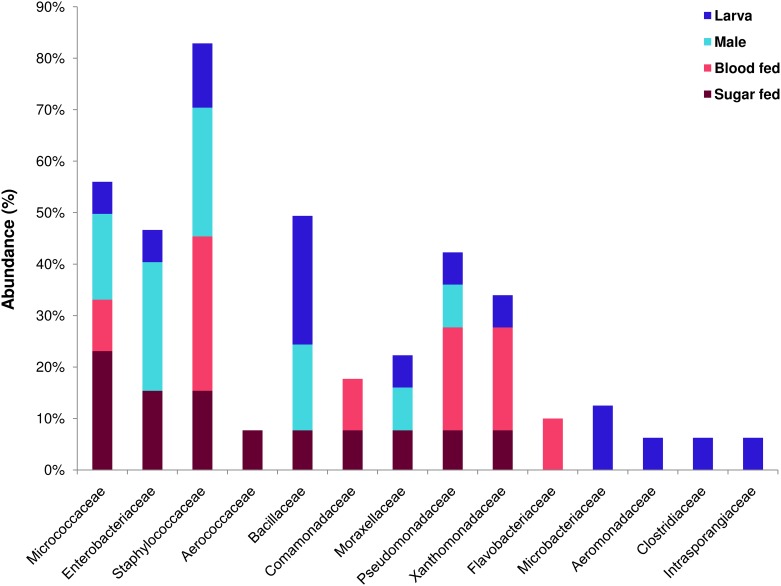
Abundance of identified bacterial species belonging to their respective family isolated from the midgut of adult sugar fed female, blood fed female, male and larvae of *Aedes albopictus*.

**Fig 4:**
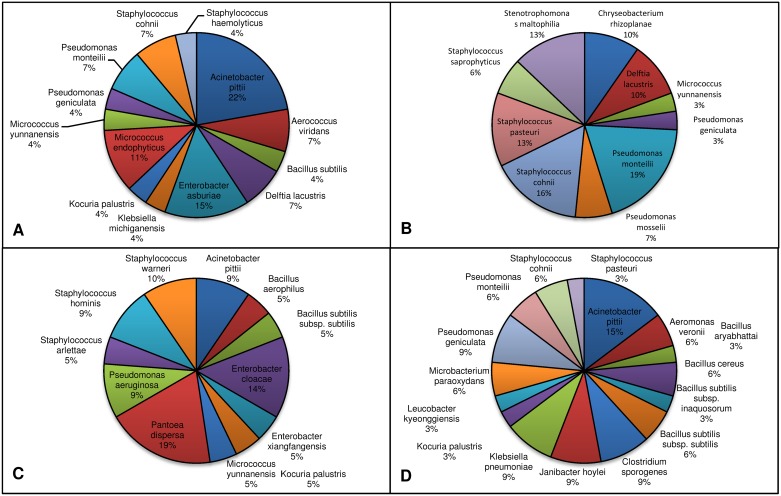
Occurrence of identified bacterial species from the midgut of A. sugar fed female, B. blood fed female, C. male and D. larvae *Ae*. *albopictus*.

A total of 13 different bacterial species from 10 genera were identified from the midgut of sugar fed *Ae*. *albopictus*, which belonged to three major phyla namely, Proteobacteria (46.15%), Firmicutes (30.77%) and Actinobacteria (23.08%). Among the phylum Proteobacteria, bacterial species belonging to the class Gamma Proteobacteria was dominant (38.46%), followed by Beta Proteobacteria (7.69%) (Figs 1 and 2). All the bacterial species of sugar fed individuals were classified under nine different families in which Micrococcaceae (23.08%) was the most abundant, followed by Enterobacteriaceae, Staphylococcaceae, Aerococcaceae, Bacillaceae, Comamonadaceae, Moraxellaceae, Pseudomonadaceae, and Xanthomonadaceae (Fig 3). Among all bacterial isolates from the sugar fed mosquitoes, *Acinetobacter pittii* was the dominant species followed by *Enterobacter asburiae*, *Micrococcus endophyticus*, *Aerococcus viridians*, *Staphylococcus cohnii*, *Pseudomonas monteilii*, *Delftia lacustris*, *Kocuria palustris*, *Micrococcus yunnanensis*, *Bacillus subtilis*, *Staphylococcus haemolyticus*, *Klebsiella michiganensis* and *Pseudomonas geniculata* (Fig 4A).

#### Bacterial isolates from midgut of blood fed *Ae*. *Albopictus*

A total of ten different bacterial species from six genera were isolated and identified from the midgut of blood fed *Ae*. *albopictus*, which belonged to four phyla, Proteobacteria (50.00%), Firmicutes (30.00%), Actinobacteria (10.00%) and Bacteroidetes (10.00%). As in sugar fed individuals, Firmicutes was the second largest phylum with three representative species. Among the phylum Proteobacteria, Gamma Proteobacteria (40.00%) was the dominant class, followed by Beta Proteobacteria (10.00%). Only single species of *Micrococcus yunnanensis* and *Chryseobacterium rhizoplanae* were identified from Phyla Actinobacteria and Bacteroidetes, respectively (Figs 1 and 2). All the bacterial species identified in the blood fed individuals belonged to 6 different families, in which Staphylococcaceae (30.00%) constituted most of the species, followed by Pseudomonadaceae, Xanthomonadaceae, Comamonadaceae, Flavobacteriaceae and Micrococcaceae (Fig 3). Two genera *Staphylococcus* and *Pseudomonas* were dominant, accounting for 60% species, rest belonged to *Micrococcus*, *Chryseobacterium*, *Stenotrophomonas* and *Delftia*. Among all the bacterial isolates, *Pseudomonas monteilii* was the dominant bacterial species followed by *Staphylococcus cohnii*, *Staphylococcus pasteuri*, *Chryseobacterium rhizoplanae*, *Stenotrophomonas maltophilia*, *Delftia lacustris*, *Staphylococcus saprophyticus*, *Pseudomonas mosselii*, *Micrococcus yunnanensis* and *Pseudomonas geniculata* (Fig 4B).

#### Bacterial isolates from midgut of male *Ae*. *Albopictus*

A total of 12 bacterial species of eight distinct genera were identified from male adult mosquitoes. Similar to the sugar fed mosquitoes, all isolates belonged to three phyla, namely Proteobacteria (41.67%), Firmicutes (41.67%), and Actinobacteria (16.67%). However, unlike sugar fed mosquitoes, in the male individuals, about 83.33% species (10 out of 12 species) belonged to only two major Phyla Proteobacteria and Firmicutes. Only two bacterial species, *Micrococcus yunnanensis* and *Kocuria palustris* were represented by phylum Actinobacteria (Figs 1 and 2). Similar to the blood fed individuals, the bacterial species of the adult males were also classifiable within 6 different families, in which the Enterobacteriaceae (25.00%) and Staphylococcaceae (25.00%) were the most abundant, followed by representatives from four different families namely Bacillaceae, Micrococcaceae, Moraxellaceae and Pseudomonadaceae (Fig 3). With three different species, genus *Staphylococcus* (25.00%) was most abundant, followed by *Bacillus* (16.67%) and *Enterobacter* (16.67%). Of the eight genera, these three genera cover about 58.33% species and rest 41.67% species belonging to five other genera. Among all isolated bacterial species, the *Pantoea dispersa* was dominant followed by *Enterobacter xiangfangensis*, *Staphylococcus warneri*, *Staphylococcus hominis*, *Pseudomonas aeruginosa*, *Acinetobacter pittii*, *Micrococcus yunnanensis*, *Kocuria palustris*, *Bacillus subtilis*, *Staphylococcus arlettae*, *Bacillus aerophilus* and *Enterobacter asburiae* (Fig 4C).

#### Bacterial isolates from midgut of larvae of *Ae*. *Albopictus*

Among all categories of individuals, maximum numbers of species (16 species) were identified from larvae of *Ae*. *albopictus*. A total of 34 bacterial isolates were identified from larvae samples, which belonged to 11 different genera under three phyla, Proteobacteria, Firmicutes and Actinobacteria. Unlike in other categories, in larvae, phylum Firmicutes was the most prominent accounting for 7 of 16 (43.75%) bacterial species belonging to two different classes: *Bacilli* (37.5%) and *Clostridia* (6.25%). Phylum Proteobacteria was the second largest phylum with five species (31.25%). With four species (25.00%), phylum Actinobacteria was the least abundant phylum (Figs 1 and 2). Total identified bacterial species were classified in 11 different families and Bacillaceae (25%) with 4 species was the most abundant, followed by Microbacteriaceae, Staphylococcaceae, Pseudomonadaceae, Aeromonadaceae, Clostridiaceae, Enterobacteriaceae, Intrasporangiaceae, Micrococcaceae, Moraxellaceae and Xanthomonadaceae (Fig 3). Among 11 different genera, *Bacillus* was the largest genera (25.00% species) followed by *Staphylococcus* (12.5%) and *Pseudomonas* (12.5%). Remaining eight genera were represented by single bacterial species. In larvae, *Acinetobacter pittii* (14.7%) was dominate bacterial species followed by *Janibacter hoylei*, *Clostridium sporogenes*, *Pseudomonas geniculata*, *Klebsiella pneumoniae*, *Microbacterium paraoxydans*, *Bacillus subtilis subsp*. *subtilis*, *Bacillus cereus*, *Staphylococcus cohnii*, *Pseudomonas monteilii*, *Aeromonas veronii*, *Leucobacter kyeonggiensis*, *Kocuria palustris*, *Bacillus aryabhattai*, *Bacillus subtilis subsp*. *inaquosorum* and *Staphylococcus pasteuri* (Fig 4D).

### Statistical analysis

Bacterial species prevalence in all the four groups of *Ae*. *albopictus* individuals were compared using Friedman test. No statistically significant difference in bacterial species prevalence between the sugar fed, blood fed, adult male and larval samples were observed (p = 0.62). Biodiversity indices like Simpson, Shannon, Evenness, Good's coverage of total isolated bacteria from different individuals were estimated and summarized in Table 1. In the present study, the value of Simpson’s diversity index ranged from 0.87 to 0.92 (maximum in larvae and minimum in blood fed individuals). The value of Shannon diversity index ranged from 2.17 to 2.66 (maximum in larvae and minimum in blood fed individuals), indicating an intermediate level of diversity. The values of evenness in this study were from 0.82 (Sugar fed) to 0.89 (larvae). The maximum value of Good's coverage was recorded from the blood fed *Ae*. *albopictus* (93.55) and minimum from male individuals (71.43).

**Table 1:** Diversity indices, total taxa, and Good’s coverage of midgut bacterial isolates of *Ae*. *albopictus* from different life stages.

	Sugar fed	Blood fed	Male	Larvae
Number of identified bacterial species in their respective category	13	10	12	16
Total number of bacteria isolated (N)	27	31	21	34
Bacterial species represented by single isolate (n)	6	2	6	5
Dominance	0.11	0.13	0.11	0.08
Simpson	0.89	0.87	0.89	0.92
Shannon	2.37	2.17	2.36	2.66
Evenness	0.82	0.87	0.88	0.89
Good's coverage	77.78	93.55	71.43	85.29

## Discussion

*Aedes aegypti* and *Ae*. *albopictus* are important vectors, and are responsible for transmission of arboviruses, globally [40]. Specifically, *Ae*. *albopictus* mosquitoes have been demonstrated to rapidly expand their territory, worldwide [41]. Due to its sturdy nature and tolerance to a broader temperature range, it is an ideal species for different climatic conditions [42]. The midgut microbiota has been shown to affect the host-pathogen interaction, ultimately influencing the potency of disease transmission of the vectors [43–47]. In this study, we aimed to explore the varying diversity of midgut microbiota of *Ae*. *albopictus* at different stages of the life cycle.

In this study, using a combination of culture and 16S rRNA sequence based methods, we could isolate and identify a total of 113 midgut bacterial isolates, belonging to four major phyla of bacteria, namely Proteobacteria, Firmicutes, Actinobacteria and Bacteroidetes. Interestingly, representatives from phyla Proteobacteria, Firmicutes, and Actinobacteria could be isolated from all the life stages of the sampled individuals, but bacterial species belonging to the phylum Bacteroidetes were isolated from the blood fed mosquitoes only.

It is thought that the midgut bacteria is generally acquired through vertical inheritance as well as from the surrounding environments [11,17,48–49]. Recently, Buck and colleagues, based on their studies, proposed that the diversity of mosquito-associated microbiota is a reflection of acquisition through various environments [49]. Additionally, diversity of the midgut microbiota is known to vary according to the life stages of the mosquitoes [11]. A large number of bacterial genera identified in this study, such as *Acinetobacter*, *Microbacterium*, *Micrococcus*, *Stenotrophomonas*, *Klebsiella*, *Pseudomonas*, *Enterobacter*, *Aeromonas*, *Clostridium* and *Bacillus* have already been reported to be common in environments, where mosquitoes breed and have also been reported to be ingested by the larvae and passed on to the adults [19,50,51]. Moreover, additional bacterial inoculation of the midgut could happen at the adult stages through the horizontal transfer from breeding sites [10,51].

Among the bacterial species isolated from the larvae, a number of species were common to adult mosquitoes, such as *A*. *pittii*, *B*. *subtilis subsp*. *subtilis*, *K*. *palustris*, *P*. *geniculata*, *P*. *monteilii*, *S*. *cohnii* in the sugar fed adult females, *P*. *geniculata*, *P*. *monteilii*, *S*. *cohnii*, *S*. *pasteuri* in blood fed adult females while *A*. *pittii*, *B*. *subtilis subsp*. *subtilis*, *K*. *palustris* in the adult males. However, more than half of the species such as *A*. *veronii*, *B*. *aryabhattai*, *B*. *cereus*, *B*. *subtilis subsp*. *inaquosorum*, *C*. *sporogenes*, *J*. *hoylei*, *K*. *pneumonia*, *L*. *kyeonggiensis*, *M*. *paraoxydans* were specific to larval individuals. This difference in the midgut microbiota diversity at different life stages may be attributable to a complex interplay between the environment and feeding habits, which in turn plays an important role in the metabolism and development of the life stages [11,17,28,49,52].

Of the bacterial isolates identified in the present study, several genera such as the *Enterobacter*, *Klebsiella*, *Pantoea*, *Acinetobacter*, *Pseudomonas*, *Bacillus*, *Staphylococcus*, *Micrococcus*, and *Aeromonas*, have commonly been isolated from the midgut of different mosquito species [17,18,21,27,28,31,52–61]. Specifically, *A*. *pittii*, *A*. *veronii*, *B*. *aerophilus*, *B*. *aryabhattai*, *B*. *cereus*, *E*. *asburiae*, *E*. *cloacae*, *E*. *xiangfangensis*, *K*. *michiganensis*, *K*. *pneumoniae*, *M*. *yunnanensis*, *P*. *dispersa*, *P*. *aeruginosa*, *P*. *geniculata*, *P*. *monteilii*, *P*. *mosselii*, *S*. *hominis*, and *S*. *maltophilia* identified in the present study have also been isolated from the midgut of *Ae*. *aegypti* and *Ae*. *albopictus*, collected from a geographically distant location during in our previous study [30], suggesting that many of the constituent microbes have established themselves as commensal, and have important roles in vector life cycle [31,52,62].

Apart from the above mentioned bacterial species, during the study we isolated *Leucobacter kyeonggiensis*, *Janibacter hoylei*, *Chryseobacterium rhizoplanae*, *Microbacterium paraoxydans*, *Clostridium sporogenes*, which, to the best of our knowledge, have not been previously reported from mosquito’s midgut. *L*. *kyeonggiensis* is a Gram-positive bacterium and was reported by Kim and Lee in 2011, as a novel species from dye waste water in Korea [63]. Although, till date *L*. *kyeonggiensis* has not been reported in insect gut, but a related species, *L*. *holotrichiae* was very recently reported from the gut of the scarab beetle larvae [64]. Another isolate, *J*. *hoylei*, a Gram-positive bacterium was identified and reported by Shivaji and colleagues, and was isolated from atmospheric samples, collected at very high altitudes (27–41 km) India [65]. *C*. *rhizoplanae*, a Gram-negative bacterial species, isolated from present blood fed individuals, was identified by Kämpfer and colleagues from rhizoplane of maize [66]. *M*. *paraoxydans* (isolated from larvae in this study) was initially reported in an acute lymphoblastic leukemia patient from Belgium and has subsequently been reported from clinical samples, as well as from fishes [67,68,69]. The bacterial species *C*. *sporogenes* (isolated from larvae in the present study) was first isolated from human faeces [70], and was subsequently reported from the gastrointestinal tract of the human and other mammalians [71–73].

In addition to other factors, the midgut microbiota diversity has also been shown to be related to the genders of the mosquitoes [17,20]. The results of our study tend to support this observation too. We could identify a number of bacterial species in the female mosquitoes exclusively (*A*. *viridians*, *D*. *lacustris*, *C*. *rhizoplanae E*. *asburiae*, *K*. *michiganensis*, *M*. *endophyticus*, *P*. *mosselii*, *S*. *haemolyticus* and *S*. *maltophilia*) and some other bacterial species specifically from males (*B*. *aerophilus*, *E*. *cloacae*, *E*. *xiangfangensis*, *P*. *dispersa*, *P*. *aeruginosa*, *S*. *arlettae*, *S*. *hominis*, *S*. *warneri*). It is well known that the midgut microbiota plays an important role in the digestion of food [57,74–75]. Males solely depend upon plant sugars [52]. On the other hand, in addition to plant sugars, female adults require blood meal for development of their ovaries, signifying a shift from carbohydrate rich diet to protein rich diet [23–26]. This shift consequently results in increased levels of enteric bacteria, while reduction in the overall microbiota diversity [28,75–76]. In our present study too, we documented the least diversity of midgut microbiota in the blood fed individuals, as compared to all the other category of individuals, supporting the previous findings.

The biodiversity index is used to quantify the number of different individuals and their distribution in any community. Simpson index is used to measure the probability of randomly drawn species of any two individuals from infinitely large community of different species [31,37]. The Simpson index is directly proportional to diversity. In the present study, the value of Simpson’s diversity index ranged from 0.87 to 0.92 (maximum in larvae and minimum in blood fed individuals). Another widely used index for comparing the diversity between various habitats is the Shannon and the values ranged from 1.5 to 3.5. Values greater than 3, indicate rich and stable diversity of habitat, whereas values less than 1.5, indicate unstable diversity, due to the pollution and degradation of habitat structure [77]. In the present study, the value of Shannon diversity index ranged from 2.17 to 2.66 (maximum in larvae and minimum in blood fed individuals). In the previous study, lowest bacterial diversity was calculated from the blood-fed mosquitoes [28] and the value of Simpson and Shannon index in our study indicate the minimum bacterial diversity from the blood fed individuals and maximum from larvae individuals. Evenness index is used to estimate the closeness of the species and determines how well they are evenly distributed among any habitat. The values of evenness in the present study ranged from 0.82 (Sugar fed) to 0.89 (larvae), which indicated that bacterial species were evenly distributed among the larvae individuals as compared to other categories of individuals. The maximum value of Good's coverage was recorded from blood fed *Ae*. *albopictus* (93.55) and lowest from male individuals (71.43), indicating that an additional 6 and 29 operational taxonomic units (OTUs) would be found if 100 additional colonies were sequenced in these two categories [78].

It is now well understood that midgut microbiota of any species is involved in various important function including digestion of food, development, providing immunity etc [11,61–62,74–75,79–80]. In vectors, specifically, midgut bacterial populations have also been shown to be involved in host-parasites interaction, resulting changes in the vectorial capacity [11,31,52,79]. Recent evidences also suggest a prominent role of the midgut microbiota in modulating the sporogonic development of parasites, augmentation of immunity against invading parasites, resulting in altered vectorial capacity [27,46,59–60,79–80]. In case of *Aedes* mosquitoes, susceptibility to Dengue viruses has been demonstrated to increase significantly in the presence *Aeromonas culicicola* and *Escherichia coli in* the midgut [48,81], while *Serratia odorifera* has been shown to enhance the susceptibility of *Aedes mosquitoes* to Dengue and Chikungunya viruses [48,82]. Interestingly, a recent study showed three bacteria isolated from *Ae*. *albopictus i*.*e*, *Enterobacter ludwigii*, *Pseudomonas rhodesiae*, and *Vagococcus salmoninarium* inhibit La Crosse virus *in vitro*, suggesting an anti-viral effect of these bacterial species [83].

In the recent years, the interest in midgut-associated bacteria has increased manifolds. Although many studies have been done on different mosquito species, diversity, functions and genetic potential of bacteria associated with *Aedes* mosquitoes is poorly understood. In recent years the population density and geographic expansion of *Aedes* mosquitoes has increased worldwide, posing significant threat of transmitting viruses such as Dengue, Chikugunya, Zika etc. Therefore, the understanding of diversity of symbiotic bacteria is essential for better understanding the adaptation and vectorial capacity, as well as to develop effective vector management strategies. The midgut microbial flora of the mosquitoes is a dynamic niche changing rapidly with the development of the vectors. Thus, understanding of bacterial communities at different life stages of vectors is important to control the vectors at different developmental stages. The findings of our present work gives a glimpse of the midgut associated microbiota of the Asian Tiger mosquito *Ae*. *albopictus*, and this information could be helpful in investigating disease transmission, and control of disease outbreaks.

## Supporting Information

S1 Table Bacterial isolates based on 16S rRNA gene sequences, %identity to NCBI/ EZtaxon and its taxonomical affiliation. (PDF)
