# Supplementary material for: Diversity of Cultivable Midgut Microbiota at Different Stages of the Asian Tiger Mosquito, Aedes albopictus from Tezpur, India
Source: PLoS One. 2016 Dec 12;11(12):e0167409. doi: 10.1371/journal.pone.0167409 (PMC5152811; doi:10.1371/journal.pone.0167409)
Supplement: S1 Table — (PDF) [file pone.0167409.s001.pdf]

S1 Table: Bacterial isolates based on 16S rRNA gene sequences, %identity to NCBI/ EZtaxon and its taxonomical affiliation

Phylum	Class	Order	Family	Bacterial species	Isolates	% Identity		GenBank accession
						NCBI	EZtaxon	
Actinobacteria	Actinobacteria	Micrococcales	Intrasporangiaceae	*Janibacter hoylei*	DRLL28	100	100	KU550177
			Microbacteriaceae	*Leucobacter kyeonggiensis*	DRLL5	99	99.7	KU550174
				Microbacterium paraoxydans	DRLL9	100	99.93	KU550178
			Micrococcaceae	*Micrococcus yunnanensis*	DRLB26	100	100	KU550157
				Micrococcus yunnanensis	DRLM11	100	100	KU550165
				Kocuria palustris	DRLM21	100	100	KU550168
				Kocuria palustris	DRLL21	100	100	KU550183
				Kocuria palustris	DRLS3	100	100	KU550135
				Micrococcus endophyticus	DRLS7	99	99.7	KU550136
				Micrococcus yunnanensis	DRLS9	100	99.7	KU550137
Bacteroidetes	Flavobacteria	Flavobacteriales	Flavobacteriaceae	*Chryseobacterium rhizoplanae*	DRLB18	99	99.49	KU550156
Firmicutes	Bacilli	Lactobacillales	Aerococcaceae	*Aerococcus viridans*	DRLS10	100	100	KU550138
		Bacillales	Bacillaceae	*Bacillus subtilis*	DRLS12	100	99.93	KU550139
				Bacillus subtilis subsp. subtilis	DRLM1	100	99.93	KU550158
				Bacillus aerophilus	DRLM7	100	100	KU550163
				Bacillus aryabhattai	DRLL2	100	99.93	KU550171

				Bacillus subtilis subsp. subtilis	DRLL4	99	99.93	KU550173
				Bacillus cereus	DRLL6	100	100	KU550175
				Bacillus subtilis subsp. inaquosorum	DRLL7	100	100	KU550176
			Staphylococcaceae	*Staphylococcus haemolyticus*	DRLS13	100	100	KU550140
				Staphylococcus cohnii	DRLS15	100	100	KU550141
				Staphylococcus saprophyticus	DRLB4	100	100	KU550151
				Staphylococcus pasteuri	DRLB15	100	99	KU550153
				Staphylococcus cohnii	DRLB9	100	100	KU550154
				Staphylococcus arlettae	DRLM3	100	100	KU550159
				Staphylococcus warneri	DRLM4	100	99.93	KU550160
				Staphylococcus hominis	DRLM12	100	100	KU550161
				Staphylococcus cohnii	DRLL14	100	100	KU550180
				Staphylococcus pasteuri	DRLL19	100	100	KU550182
	Clostridia	Clostridiales	Clostridiaceae	*Clostridium sporogenes*	DRLL25	99.78	99	KU550172
Proteobacteria	Beta Proteobacteria	Burkholderiales	Comamonadaceae	*Delftia lacustris*	DRLS27	100	100	KU550147
				Delftia lacustris	DRLB5	100	100	KU550152
	Gamma Proteobacteria	Aeromonadales	Aeromonadaceae	*Aeromonas veronii*	DRLL34	99.86	99	KU550184
		Enterobacteriales	Enterobacteriaceae	*Klebsiella michiganensis*	DRLS16	99	100	KU550142
				Enterobacter asburiae	DRLS23	99	99.85	KU550145

				Pantoea dispersa	DRLM15	100	100	KU550164
				Enterobacter xiangfangensis	DRLM20	99	99.62	KU550166
				Enterobacter cloacae	DRLM10	99	99.79	KU550167
				Klebsiella pneumoniae	DRLL24	99.79	99	KU550185
		Pseudomonadales	Moraxellaceae	*Acinetobacter pittii*	DRLS19	100	100	KU550144
				Acinetobacter pittii	DRLM18	100	100	KU550169
				Acinetobacter pittii	DRLL11	100	100	KU550179
			Pseudomonadaceae	*Pseudomonas monteilii*	DRLS18	100	100	KU550143
				Pseudomonas mosselii	DRLB1	100	99.93	KU550148
				Pseudomonas monteilii	DRLB12	100	100	KU550155
				Pseudomonas aeruginosa	DRLM5	100	100	KU550162
				Pseudomonas monteilii	DRLL1	100	100	KU550170
		Xanthomonadales	Xanthomonadaceae	*Pseudomonas geniculata*	DRLS26	100	100	KU550146
				Pseudomonas geniculata	DRLB2	100	100	KU550149
				Pseudomonas geniculata	DRLL16	100	100	KU550181
				Stenotrophomonas maltophilia	DRLB3	100	99.78	KU550150

Where: DRLS- Sugar fed female mosquitoes, DRLB- Blood fed female mosquitoes, DRLM- Male mosquitoes, DRLL- Larvae
